# The role of older patients’ goals in GP decision-making about medicines: a qualitative study

**DOI:** 10.1186/s12875-020-01347-y

**Published:** 2021-01-08

**Authors:** Kristie Rebecca Weir, Vasi Naganathan, Stacy M. Carter, Chun Wah Michael Tam, Kirsten McCaffery, Carissa Bonner, Debbie Rigby, Andrew J. McLachlan, Jesse Jansen

**Affiliations:** 1 Sydney School of Public Health, The University of Sydney, Sydney, New South Wales 2006 Australia; 2 Wiser Healthcare, Sydney School of Public Health, The University of Sydney, Sydney, NSW 2006 Australia; 3 Centre for Education and Research on Ageing (CERA), Concord Clinical School, Faculty of Medicine and Health, The University of Sydney, Sydney, New South Wales 2006 Australia; 4 Ageing and Alzheimer’s Institute, Concord Repatriation General Hospital, Concord West, New South Wales 2139 Australia; 5 Australian Centre for Health Engagement, Evidence and Values (ACHEEV), School of Health and Society, Faculty of the Arts, Humanities and Social Sciences, University of Wollongong, Keiraville, New South Wales 2522 Australia; 6 Primary and Integrated Care Unit, South Western Sydney Local Health District, Liverpool, New South Wales 2170 Australia; 7 School of Population Health, The University of New South Wales, Sydney, New South Wales 2052 Australia; 8 School of Clinical Sciences, Faculty of Health, Queensland University of Technology, Brisbane City, Queensland 4000 Australia; 9 Faculty of Medicine and Health, Sydney Pharmacy School, The University of Sydney, Sydney, New South Wales 2006 Australia

**Keywords:** Deprescribing, Shared decision-making, Communication, Polypharmacy, Multimorbidity, Complexity, Older adults, Primary care, Goals

## Abstract

**Background:**

To optimise medication use in older people, it is recommended that clinicians evaluate evidence on potential benefits and harms of medicines in light of the patients’ overall health, values and goals. This suggests general practitioners (GPs) should attempt to facilitate patient involvement in decision-making. In practice this is often challenging. In this qualitative study, we explored GPs’ perspectives on the importance of discussing patients’ goals and preferences, and the role patient preferences play in medicines management and prioritisation.

**Methods:**

Semi-structured interviews were conducted with GPs from Australia (*n* = 32). Participants were purposively sampled to recruit GPs with variation in experience level and geographic location. Transcribed audio-recordings of interviews were coded using Framework Analysis.

**Results:**

The results showed that most GPs recognised some value in understanding older patients’ goals and preferences regarding their medicines. Most reported some discussions of goals and preferences with patients, but often this was initiated by the patient. Practical barriers were reported such as limited time during busy consultations to discuss issues beyond acute problems. GPs differed on the following main themes: 1) definition and perception of patients’ goals, 2) relationship with the patient, 3) approach to medicines management and prioritisation. We observed that GPs preferred one of three different practice patterns in their approach to patients’ goals in medicines decisions: 1) goals and preferences considered lower priority – ‘Directive’; 2) goals seen as central – ‘Goal-oriented’; 3) goals and preferences considered but not explicitly elicited – ‘Tacit’.

**Conclusions:**

This study explores how GPs differ in their approach to eliciting patients’ goals and preferences, and how these differences are operationalised in the context of older adults taking multiple medicines. Although there are challenges in providing care that aligns with patients’ goals and preferences, this study shows how complex decisions are made between GPs and their older patients in clinical practice. This work may inform future research that investigates how GPs can best incorporate the priorities of older people in decision-making around medicines. Developing practical support strategies may assist clinicians to involve patients in discussions about their medicines.

**Supplementary Information:**

The online version contains supplementary material available at 10.1186/s12875-020-01347-y.

## Background

Managing medications in older people can be challenging for general practitioners. Medication use is often problematic because of over-prescribing, under-prescribing, inappropriate selection of a medication and avoidable adverse drug reactions [1, 2]. Further to this, specific classes of medications and polypharmacy (taking 5 or more medications) can be harmful [3]. It is important to ensure that the benefits of starting or continuing a medication outweigh the harms, and that they support the larger aims of the person’s life and impose the smallest possible burden [4, 5]. One way to reduce medication burden is by deprescribing through dose reduction or discontinuing selected medicines, when *harms outweigh benefits within the context of a patient’s care goals, level of functioning, life expectancy, values and preference*s [6]. Deprescribing is now considered an essential element of *good* prescribing [7]. Clinicians and older patients face complex trade-offs and considerable uncertainty, therefore many treatment decisions in this context are preference-sensitive [8]. In order to optimise medicines for this group, patient-centred communication and shared decision-making (SDM) are essential [9, 10].

SDM is a process during which clinicians and patients make health decisions collaboratively, considering evidenced-based information and preferences of the patient [11]. Informing, eliciting and helping patients construct their preferences is a core aspect of shared decision-making [12]. For many older patients with polypharmacy these are challenging decisions as there are so many factors to take into account including multi-morbidity, transitions of care, frailty and limited life expectancy. Although incorporating patient goals and preferences into treatment decisions can improve health outcomes [13], elicitation and alignment of patient preferences with medical decisions is arguably the most difficult part of SDM [14]. In recent years there has been a push for goals and preferences to be considered an essential part of SDM, acknowledging this step has been somewhat overlooked in the SDM academic literature [15, 16]. This highlights the importance of eliciting patients’ goals and preferences to inform complex decisions faced by an older person with comorbidities taking multiple medicines [17, 18].

Accounting for a person’s goals and preferences into decisions about medicines are a key element of prescribing and deprescribing algorithms [6], conducting a medication review [19], and features in the literature on patient-centred approaches to medicines use [20]. Conceptually, person/patient centred care (PCC) emphasises the moral importance of responsiveness to individuals and what they value [21]. This is not to mean patient choice should be mandated or that health professionals must agree with and do whatever a patient says they want. Rather it supports the idea that *“patients’ personal preferences be taken seriously, but in a nuanced and situation-sensitive way”* [21]. Patient input is valuable as they are the expert in their experiences and how they feel about their medicines. Interpersonal relationships and professional support are paramount to ensure that individuals can engage in and influence their care [22]. Individuals can then feel supported to be involved at the level they are comfortable and capable with. In practice, however, clinicians find it challenging to facilitate patient involvement in decision-making [23].

Clinician and patient factors have previously been identified as barriers to involving older patients in decisions about medicines [24]. Clinicians may believe they know their patient’s preferences already or incorrectly assume an older person cannot contribute to decisions about their medicines [25, 26]. Older people have varied preferences for involvement in health decisions, but most older people want their perspectives heard [10]. For some older people inadequate health literacy skills, physical problems (e.g. poor hearing) or cognitive impairment makes it challenging to be involved in decisions about medicines [27–29]. In addition, there is an interplay of complex factors that lead an individual to accept polypharmacy or resist deprescribing, such as knowledge about medicines and trust between the patient and clinician [10].

Communication strategies facilitate decision-making aligned with preferences and goals for persons with serious illness or near the end-of-life [30, 31]. However, there is little guidance for clinicians on *how* to talk to older people about their goals and preferences and *how* to combine a persons’ priorities with the clinical aspects of decision-making about medicines. To advance this area it is important to first have a greater understanding of the role of goals and preferences in decision-making about medicines made by GPs’ and their patients in everyday clinical practice [32]. This study will explore GPs’ perspectives on the importance of discussing a patient’s goals and preferences, and incorporating these in decisions about their medicines.

## Methods

### Design

This qualitative study used face-to-face or phone/video call interviews with 32 GPs from Australia. See Supplementary file A: Consolidated criteria for reporting qualitative research (COREQ) for more detail.

### Data collection

A semi-structured interview schedule was developed in conjunction with a multidisciplinary research team, which included experts in geriatrics (V.N.), general practice (L.T.), pharmacy (A.M., D.R.), epidemiology (L.I.), ethics (S.C.), health psychology (J.J., K.M., C.B.), qualitative research methodology (K.W., S.C., J.J., K.M., C.B.), and a consumer representative (J.C.). Topics included GPs’ views on the role of patients’ goals and preferences in medicines management including deprescribing and GPs’ experiences of medication reviews. GPs’ views on medication reviews are reported separately to enable a sufficiently detailed description of the study findings [33].

The interview schedule was piloted with two GPs and modified. Interviews lasted between 24 and 55 min, and de-identified audio-recordings were transcribed verbatim. Between February and October 2017, interviews were conducted by one researcher (K.W).

GPs were asked about if/how they communicate with older patients about polypharmacy. Basic demographic data were collected, including gender, age, years of GP practice (Table 1).

**Table 1:** Characteristics of General Practitioners (GPs)

GP characteristics	No. of GPs ***n*** = 32
Years of GP working experience (years)	
1–9	14
10–19	6
20–29	4
30+	8
Gender	
Female	18
Male	14
Role at medical practice	
Registrar/in training	7
Contractor/sessional/salaried	17
Principal/partner	8
Number of GPs at medical practice	
1–5	14
6–10	9
11+	9
How many patients seen who were 75+ years (%; estimate per year)	
1–19	10
20–39	15
40+	7
Index of Relative Socio-economic Disadvantage Quintile^a^	
1 (most disadvantaged)	2
2	8
3	0
4	6
5 (least disadvantaged)	16

^a^Socio-Economic Indexes for Areas (SEIFA) by Local Government Area. 1 = most disadvantaged, 5 = least disadvantaged

### Participant recruitment

**Fig. 1:**
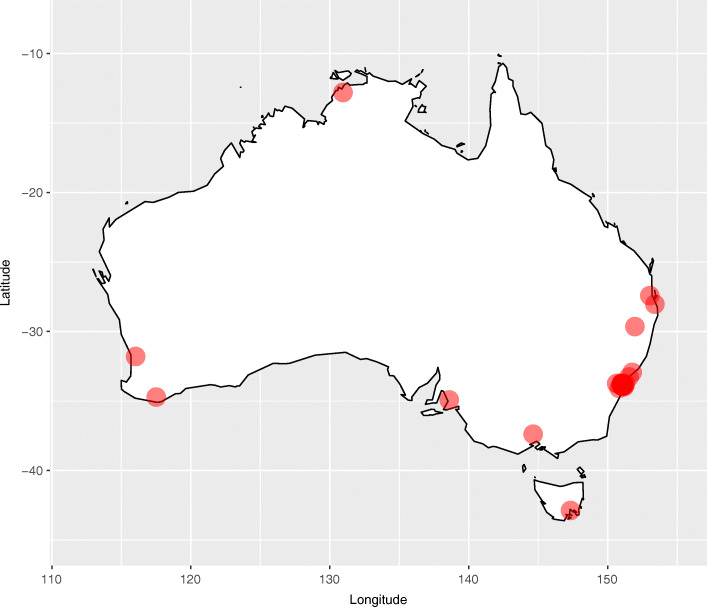
Map of Australia with clinic locations of the participants

Recruitment commenced in New South Wales, then extended to other states in Australia (Fig. 1). We used purposive sampling to recruit GPs, aiming for variation in demographic characteristics. The study was advertised via the newsletters and email lists of GP organisations (Royal Australian College of General Practitioners (RACGP), Primary Health Networks, Australian Medical Association), through professional networks and publicly available information (email invitation), in social media, and at medical conferences. Regional GPs were accessed by making telephone contact with practice managers, through colleagues, and advertising with the Aged Care Network (RACGP specific interest group). Subsequent active ‘snowballing’ was used to access hard to reach participant demographics – where participating GPs were asked to nominate their colleagues to be invited to participate. GPs provided verbal consent before participating in a face-to-face interview (17 participants) or via telephone/video conference (15 participants). Participants received $AU50 for their time.

### Analysis

Framework Analysis was used to organise the interview data and identify themes [34]. This is a matrix-based method of thematic analysis, with participants’ data summarised as rows and themes as columns [10]. Framework Analysis involves a five-step process. Firstly, one researcher (K.W.) read through a subset of transcripts to identify themes. Along with the interview schedule, these themes formed the basis for the initial coding framework, which was reviewed and discussed by qualitative researchers (K.W., I.B., J.J.). Two researchers then independently reviewed another subset of transcripts, developed codes, and compared the data for differences and similarities in the data and coding (K.W., I.B.). Researchers then discussed and together established overarching themes and categories, and developed the matrix (K.W., I.B., J.J., and K.M.). Two researchers (K.W., I.B.) divided the transcripts and independently summarised the themes and quotes from each transcript into the matrix with continuous discussion with other researchers (K.W., I.B., J.J., K.M. and V.N.). At this point, the team observed what appeared to be three distinct patterns of participants. To test this, a new subset of participants was selected, and two researchers independently categorised these participants into practice patterns; this showed that the patterns were sound and could be reliably applied to the data (K.W., V.N.). When all of the data were coded and summarised into the matrix, the framework was examined within and across themes and participants to identify further relationships and themes. An academic GP (C.T.) analysed summary data, providing a dual perspective as both a participant and researcher. Rigour was addressed throughout this process by ensuring a detailed documentation of the analysis process; repeat coding of transcripts; constant comparison of data with continuous discussion of themes.

## Results

Table 1 demonstrates diverse characteristics in the sample, including experience ranging from 1 to 50 years (average, 17 years). There were more female participants with < 10 years’ experience (13/14) and more male participants with 30+ years’ experience (7/8). Approximately half the GPs (14/32) worked in smaller practices with 1–5 GPs. GPs were recruited across Australia, including regional locations.

We developed three main practice patterns from the data. Most GPs had a preferred practice pattern, which related to their years of experience. We conceptualised a practice pattern as a way of practicing medicine in relation to older adults and polypharmacy. A practice pattern was a set of practices that tended to cluster together to form an identifiable way of doing things: this could include, for example, a way of acting, reasoning, and communicating about medicines. These patterns were not always mutually exclusive and individual GPs might move between two practice patterns, which might be as a result of gaining more experience or working in a different context. Overall, any individual GP at a specific point in time was likely to have one preferred pattern.

Our analysis presents these practice patterns individually, with key differing features discussed: 1) definition and perception of patient goals, 2) relationship with the patient, 3) approach to medicines management and prioritisation.

### Practice pattern 1 ‘directive’: goals and preferences considered a lower priority in clinical decisions

Overall, GPs with characteristics of this practice pattern appeared to encourage older people to think as a doctor would, and convince patients of what their medication goals should be. GPs who exhibited traits of this practice pattern tended to have less clinical experience and seemed to use a more directive decision-making approach. They tended to follow guidelines with a strong focus on treatment adherence, and perceived little room to tailor medication to individuals.

#### Definition/perception of goals

A feature of this practice pattern was to determine for their patients what the goals of care should be: *“they’re always like, oh my blood pressure’s fine. But it’s like, no, you need to take this for a different reason… So just explain to them why we’re doing it. And usually once you explain to them the rationale, they get it. It’s like statins as well, like if you’re no different when you’re on it, but the reason that we’re doing it is to lower your cardiovascular risk”* (ID15, female, 2 years’ experience)*.* They appeared not to have explicit discussions with their patients around what mattered to them in relation to their medicines. While GPs using this practice pattern had a sense that patients differ to some extent, and recognised the need to respond in slightly different ways, this was to convince the patient to follow recommendations: *“the patients, um, have to believe their doctors but that belief is based on how well you’re communicating all their medical problems with them”* (ID13, female, 5 years’ experience). GPs adhering to this practice pattern also understood goals of care may change based on the patient’s clinical trajectory.

When GPs who exhibited traits of this ‘Directive’ pattern were asked if they thought it was possible to get a sense of their patients’ goals and preferences, some GPs interpreted this to mean changing patient goals to better match clinician goals: *“it is sometimes difficult to marry up patient goals and clinician goals. Um, and I think that is due to health literacy as well and how well we explain things to patients when we start them on medications”* (ID27, female, 3 years’ experience). GPs spoke about advantages of talking to patients about goals to gain trust and improve compliance: *“I think it is very important. Because, their goals need to be somewhat aligned to ours for them to be compliant”* (ID27, female, 3 years’ experience). However, some GPs using the ‘Directive’ practice pattern reported that eliciting patient goals and preferences could not be prioritised because of time pressures: *“It’s probably quite important (laughs). It’s always hard to find time amongst everything else that you’re trying to do and keep up to date. Especially with older people”* (ID3, female, 9 years’ experience).

#### Relationship between GP and patient

Some GPs were hesitant to encourage patient involvement in medication decision-making. Some were trying to instil confidence and were worried that seeking the patient’s opinion might make them appear less skilled: *“Being a younger doctor, if you’re bringing up questions it seems like you’re not sure what you want to do and you’re … trying to shift responsibility on to [the patient]. So, there might be that meta-effect of them losing confidence in you as a doctor”* (ID20, female, 2 years’ experience). Some GPs viewed older patients as preferring a passive role in decisions: *“A lot of patients … they’re quite happy to do what you say”* (ID10, female, 4 years’ experience). Some GPs placed the onus on patients to initiate discussions about medication goals: *“I think it comes down to insight. And how much … um, they’re willing to take responsibility for their own health. Like there are people who are all over it and know what they want, their priorities, etc … I think those that don’t want to take responsibility of their health problems do have … that’s where the issues are”* (ID15, female, 2 years’ experience).

#### Medicines management and prioritisation

Most GPs utilising the ‘Directive’ practice pattern believed their older patients were taking necessary medications, perceiving limited flexibility to make changes based on patient preferences: *“I would say the majority of cases you can’t really, there’s not really anything that you can cut out, but we do have that discussion from time to time”* (ID10, female, 4 years’ experience). In contrast to other practice patterns, GPs preferring this pattern said it could be difficult to achieve adherence: *“it’s like to prove to them this is why it’s necessary”* (ID15, female, 2 years’ experience). GPs tended to focus on following clinical guidelines and improving medication safety and adherence. Often this involved convincing patients to take their medicines: *“taking the medication is going to improve your life expectancy, but it’s going to come with some little bit of side effects. That we have to put up with* (ID13, female, 5 years’ experience).

Patient preferences sometimes led to medication-related decisions, e.g. for low risk/low gain medications or if the patient reported experiencing a side effect. However, if a medication was prescribed by a specialist or hospital clinician, GPs preferring this practice pattern felt they could not make changes: *“the patient I guess is at the mercy of the specialists, um, in terms of the more complex medications”* (ID27, female, 3 years’ experience). They reviewed their patient’s medications to keep on top of changes: *“If I’m not running late then I’ll always go through the list of medications … usually there’s at least one or two medications that they’re no longer on or has been changed by the specialist …*” (ID11, female, 4 years’ experience).

Some GPs discussed attempting to deprescribe medications with mixed results. GPs tried to explain a continuum or categorisation of older patients in order to understand their willingness to deprescribe: *“you can get that sense of feeling that [patients are] medication oriented or whether they’re happy to quit, stop medications”* (ID13, female, 5 years’ experience). Barriers to deprescribing included concern about patient harm, criticism from the patient, colleagues or specialists, or a specialist restarting the medication without consultation. Patient resistance to deprescribing was also reported: *“often they believe they should be on most of them”* (ID27, female, 3 years’ experience).

When being ‘Directive’, GPs utilised various services to support patient care. These are summarised in Table 2. For example, using a program for medication reviews performed by a pharmacist allowed GPs to gain further expertise and insights into relevant clinical pharmacology and to shift some tasks they would normally do (i.e. reviewing medications). This could be viewed in different ways: to improve efficiency, or a coping strategy to relieve some pressure or recognition of other peoples’ greater ability to perform a certain task. GPs explained that this made it easier to continue the discussion about goals and preferences with the patient or at least have some understanding of patient preferences in medication-related decisions: *“if someone’s done that for me that does help. That’s a useful piece of information”* (ID7, male, 3 years’ experience).

**Table 2:** List of services utilised by participants

Services/resources	Type	Who is involved
**Government services**	- Chronic care plan, management plan, indigenous health review, health assessment for patients aged 75 years and above - Medication review program	Practice nurse + GP + patient Accredited pharmacist + GP + patient
**Online resources**	- Websites on medicines safety i.e. Australian Medicines Handbook, NPS MedicineWise, MIMS. - Online risk calculator for cardiovascular disease, iconarrays to discuss risk about specific mediations. - Deprescribing websites and apps	GP
**Guidelines**	- Therapeutic guidelines, condition specific guidelines. - To find alternatives treatments	GP
**Other health professionals**	- Community pharmacists, geriatricians	GP + health professional + patient

### Practice pattern 2 ‘goal-oriented’: goals are central to decision-making about medicines

GPs who preferred this practice pattern generally used a patient-focused approach to medicines management in older patients, recognising the importance of explicitly eliciting patient goals and tailoring medications accordingly. A few GPs who exhibited traits of this practice pattern compelled patients to be involved in decisions about medicines without recognising this can be burdensome: *“[GP has a special screen so patients can see their medical record] I use a larger screen, a curved screen … [to] get patients involved, um, have some transparency … empower them with the fact that this is their health and they need to take control of that. So first getting them engaged and then having some control”* (ID2, male, 25 years’ experience). These GPs acknowledged the potential risk of polypharmacy and uncertainty around benefits of medicines in older patients, were generally confident in deviating from guidelines if needed and described positive experiences with deprescribing. The ‘Goal-oriented’ practice pattern was more commonly followed by GPs in the middle stage of their career.

#### Definition/perception of goals

GPs expressed the importance of discussing goals and preferences with all older patients: “*Everybody’s capable of … having a sense of their goals and what they want with their medication”* (ID14, female, 28 years’ experience). They described this as essential for quality care, ensuring that medications fit into and improve the patient’s life and not the other way around: “*making sure that what we’re doing is in line with what they want. So ultimately, we’re there to improve [the patient’s] health, as they see it … not to treat the numbers”* (ID26, male, 10 years’ experience). GPs who preferred this practice pattern perceived patients’ goals and preferences as individual, dynamic and changing over time. Quite a few ‘Goal-oriented’ GPs discussed this routinely, setting aside dedicated time.

#### Relationship between GP and patient

GPs applying this practice pattern expressed the importance of communicating about medications to improve knowledge and empower older patients to be actively involved in their health. They seemed to reject the discourse that ‘time-poor’ GPs are unable to meaningfully talk to their patients: *“the thing that’s less possible for most GPs is their claim that they don’t have enough time. The fact is we all have the same amount of time. So, you know, these are … er, nobody’s making GPs spend too little time with patients, they’re doing it themselves”* (ID12, male, 38 years’ experience).

A few GPs acknowledged that some older patients may not be used to involvement in medication decisions, but one GP wanted a ‘paradigm shift’: *“I want to say it’s a shared role. It’s not me telling you and you just listening to what I’m saying. I want you to interact. I want you to understand. I want you to challenge...”* (ID2, male, 25 years’ experience). Some GPs recognised the difficulties of taking a shared approach and sometimes it was their clinical responsibility to make the final decision. For example, one GP had spent months discussing the benefits and harms of stopping an anticoagulant medication (warfarin) with a frail patient, who was unable to come to a decision: *“[The patient] couldn’t make a decision about it … You know, the narrative in his mind was well don’t I need to be on this? If I don’t have this aren’t I going to have a stroke? Er, and the answer to that is yes, possibly. But he was also at the point where, you know, realistically I thought that the harms were probably getting high enough and he was falling all the time. And he was having bruises. Er, and eventually I just had to make an executive decision about that. Right, I’m stopping it”* (ID29, male, 13 years’ experience).

#### Medicines management and prioritisation

GPs adhering to the ‘Goal-oriented’ practice pattern were more likely to emphasise and be comfortable with the uncertainties of medical practice. They talked about the limitations of the evidence for medications in the older population, the potential for harm when following the guidelines and the need to see the patient as an individual: *“[Another] doctor had followed the guidelines perfectly …*. *but it just didn’t work for this man. And it didn’t fit with his amazing quality of life … And I think that that’s the hard part … doctors who either don’t know their patients well or who are new coming in, um, can be very strict at following the guidelines”* (ID24, female, 7 years’ experience).

Whilst acknowledging the challenges of deprescribing and stopping medications that took years to convince the patient to take, GPs using this practice pattern had an overwhelmingly positive attitude towards deprescribing, particularly preventive medications. Unless a patient had voiced a concern or fear of a specific condition/illness: “*[A patient’s] quality of life on a daily basis is more important than pushing all this preventative stuff, which is what a lot of, half of the medications they’re on are for really”* (ID20, female, 2 years’ experience).

GPs using the ‘Goal-Oriented’ practice pattern reported prioritising medications based on older patients’ goals and preferences. GPs perceived this as challenging but necessary: *“That’s a very complex and tricky thing to do … So it does, er, there’s no simple way to do it and it does depend on what [the patient’s] main goals are as well and what their attitude to life is and how long they want to live and so on”* (ID12, male, 38 years’ experience). These GPs seemed to consider patient input as valuable and equal to their own clinical input, for example, recognising that ‘quality of life’ is different for every patient: *“My interest mainly is quality of life for patients and asking them what … what that means to them. Taking medication for what their goals are”* (ID9, male, 10 years’ experience). This practice pattern engaged GPs who recognised that patients differ in the trade-offs they are willing to make in terms of harms and benefits of medications: “*I’ll actually go over it and say, well these are the risks and that’s how likely you’re going to have it. I mean, how important is that for you?”* (ID20, female, 2 years’ experience).

These ‘Goal-oriented’ GPs were attuned to their patient’s self-determination in several ways. They accepted if a patient decided not to take a medication – even if it was against their recommendation. GPs gave examples of complex discussions to consider patients’ goals when making medication decisions. For example, one GP had a patient diagnosed with atrial fibrillation by another clinician and prescribed a medication that controls heart rate (beta blocker). This medication caused the patient to feel breathless and fatigued to the point where they cancelled a family trip. A discussion was had between the GP, patient and patient’s daughter about the patient’s goals: *“Yes, you don’t want to have a debilitating stroke and end up in a high care nursing home, but you also won’t have quality of life now … and eventually we just put him back on what he was on before, and decided to pretend that we never found out about the atrial fibrillation”* (ID24, female, 7 years’ experience).

‘Goal-oriented’ GPs tended to tailor various services with the aim of improving care for older patients. While these services (Table 2) were utilised to support goal elicitation to develop therapeutic relationships, GPs also recognised that these services often fell short of facilitating patient-centred care.

### Practice pattern 3 ‘tacit’: goals and preferences considered in decisions around medicines but not explicitly elicited

GPs using the ‘Tacit’ practice pattern considered patient goals important to inform care. GPs inferred what mattered most to their patients based on years of experience and longstanding relationships with older patients. However, these GPs described difficulties tailoring medication management to older patients’ goals and favoured a conservative approach with a strong focus on medication safety and avoiding risks.

#### Definition/perception of goals

‘Tacit’ GPs reported they considered patients’ goals and preferences in the periphery of their clinical decisions. These GPs – many with extensive experience – felt they understood their older patients’ goals through long-standing, trusting relationships with older patients and many conversations over the years, without explicitly discussing goals: *“I’m not sure that I really ask them a lot”* (ID5, male, 31 years’ experience). A few GPs using the ‘Tacit’ practice pattern considered that most patients had broader, universal goals: *“I think [patient] goals are to have their symptoms relieved … and to live as long as possible … And that’s the universal goals of anybody. So talking about different patients having different goals, I don’t know if that’s a real-world issue”* (ID25, male, 36 years’ experience).

General patient goals identified by GPs with traits of this practice pattern tended to be a combination of staying alive and quality of life. Patient goals were expressed as: symptom relief, improving wellbeing, being pain-free, maintaining independence, taking as few medications as possible to avoid side effects. GPs contextualised patient goals in a broader understanding of ‘the human condition’ and considered this in the background of decision-making. For example, one GP’s ‘guiding principle’ was: *“To look after [patients], to love them, love them in small way, um … help them with a life that is, that is healthy and meaningful. Um, and if medications might be interfering with that then I think … er, I would, I might change that”* (ID5, male, 31 years’ experience).

#### Relationship between GP and patient

Most GPs who engaged with the ‘Tacit’ practice pattern said they made decisions for their patients for two reasons: 1) the clinician has inherent responsibility as the prescriber, with knowledge asymmetry between clinician and patient, 2) from their perspective, many older patients preferred a passive role in decisions: *“That age group usually are happy to be guided by me. You know, they just take … whatever I say they just believe”* (ID32, male, 37 years’ experience).

The proportion of older patients seen by these GPs was high and some GPs expressed fatigue in caring for patients with complex chronic conditions on a high number of medicines, visiting residential care facilities and making house calls. GPs talked about dedicating time to fully manage the issues older patients experience: “*Many of the patients I’ve seen for 30 plus years … but I also have time to talk to them, ‘cause I’m not a … 6 patients per hour kind of practice”* (ID25, male, 36 years’ experience).

A few GPs stated their profession is undervalued as the *“conductor of the orchestra”* (ID32, male, 37 years’ experience). One GP expressed frustration with patients’ relatives and transitions from hospital to an aged care facility, considering himself as overseeing patient care: *“I … invariably will reduce medications in the nursing home after the hospital attendance. Sometimes with arguments from the relatives. [The patient’s relatives say:] No, specialist put her on it. I said, well in that case ask the specialist to come and give you a prescription because I will not. Full stop. And we have arguments. Don’t want to? Change doctors... I’m, I’m quite brutal about this”* (ID31, male, 50 years’ experience).

#### Medicines management and prioritisation

GPs using the ‘Tacit’ practice pattern discussed the challenges in medicines management and prioritisation in patients who are clinically stable. GPs dichotomised medicines as essential or non-essential, with essential medicines having little flexibility for change. They discussed prioritising medications based on their clinical importance and indication: *“People are getting swollen ankles. You put them on fluid tablets but then, you know, you think well is this really worth it? ‘Cause their … swollen ankles are not going to kill them. But having to get up to go to the toilet in the middle of the night might kill them, you know. They might fall and break their hip”* (ID16, male, 40 years’ experience). ‘Tacit’ GPs were experienced in prescribing and medicines interactions, including the complex nature of competing chronic conditions and tended to avoid risks as much as possible. For example, prescribing the minimal number of medications to begin with, use of dose administration aids, maintaining the *status quo* to avoid confusion or potential adverse events, and making changes to medications only if a patient has raised an issue.

For high-risk patients, GPs tended to focus on maintaining patients living independently for as long as possible and would minimise medications that may increase the risk of an acute incident such as a fall. ‘Tacit’ GPs reported utilising various services (Table 2) that are designed to help older patients stay in their home and manage medications to assist with practical tasks whilst maintaining their independence: *“My aim is to try and keep them at home with an aged care package”* (ID32, male, 37 years’ experience). GPs using this practice pattern were aware of the delays and wait times associated with different services, thus commenced these processes early, understanding the trajectory of a patient and intervening pre-emptively.

All GPs describing characteristics of the ‘Tacit’ practice pattern had some experience with deprescribing in different ways and for different reasons. Some GPs considered deprescribing as highly important and the key to optimising medications: *“At the end of the day we’re trying to improve patient outcomes by improving medication uses. It’s not really what the patient primarily wants”* (ID16, male, 40 years’ experience). For some GPs, deprescribing wasn’t their *“guiding principle”* (ID5, male, 31 years’ experience) and was often challenging in practice due to the difficulty convincing patients to cease medications: *“If they’re really wedded to their medications it’s sometimes it’s really hard. Like the Zocor lady [105-year-old patient taking simvastatin]. Um … and I guess you pick your battles”* (ID5, male, 31 years’ experience). One GP said he appreciated if a patient’s medication were stopped in hospital, as this was less of a risk than deprescribing in general practice: *“The only time we can do it [deprescribe a medication] is when people end up in hospital for whatever reason … and then those tablets are stopped. And somehow, they survive without them, then you’re fine. But when we do it from here, then it becomes a risk”* (ID23, male, 39 years’ experience).

In general, GPs viewed prioritising medicines as their responsibility. However, occasionally, if a patient says *“I’m not achieving my decency and my dignity without those tablets”* (ID23, male, 39 years’ experience), some GPs would shift to prioritising a medication for symptom control over an ‘essential’ medication based on what is most important to the patient.

## Discussion

In this study, we identified three practice patterns displayed by GPs, varying considerably in their definition and perception of patient goals, the patient-doctor relationship and their approach to medicines management. Gaining an in-depth understanding about the perceived role of older patients’ goals and preferences provides useful knowledge about how GPs use patients’ priorities in everyday consultations.

The strengths of this study include the heterogeneous sample of GPs varying in location and years of experience. The interview topics were not geared towards promoting patient goals as an element of shared decision-making. Rather, GPs were asked to reflect on their own perceived importance of patients’ goals and if this plays out in clinical practice. Limitations include a relatively small number of GPs on which findings are based, who were willing to participate in an interview – although the sample size was sufficient for qualitative research. There was some degree of homogeneity within GPs with similar practice patterns. For instance, the GPs preferring a ‘Directive’ practice pattern were more likely to be female with fewer years of experience. These participants may represent a more motivated subset of GPs who have an interest in goal elicitation. Quantitative research could be useful to further verify these practice patterns and quantify the percentage of GPs that follow each pattern.

We feel there is value in discussing each practice pattern according to the definition, conceptualisation and implementation of patient-centred care (PCC) by Entwistle & Watt [21]. The three fundamental aspects of PCC are: 1) to treat patients with respect and compassion, 2) to be responsive to a patient’s unique identity and subjective experience, 3) to support patients to be as autonomous as they can be (Table 3).

**Table 3:** Practice patterns of GPs with the fundamental elements of patient-centred care

Practice pattern	Patient-centred care (PCC) fundamental elements
**‘Directive’**	• Followed clinical practice guidelines with a focus on medication adherence, encouraged patients to try to think in the same way a doctor would, perceived older patients prefer not to have an active role in decision-making
**‘Goal-oriented’**	• Eliciting patients’ goals and preferences seen as fundamental for providing good quality care, confident to deviate from guidelines, encouraged patients to be involved in decision-making some without consideration of the patient’s actual preferred level of involvement
**‘Tacit’**	• Patients’ goals and preferences used in an intrinsic way without necessarily asking them, prioritised avoiding risks and medication safety, assumed patients prefer their doctor to make decisions, relying on their doctor’s expertise and their longstanding relationship with the patient

For the first practice pattern ‘Directive’, GPs recognised that individual patients’ goals and preferences differed, although this was not operationalised often – effectively deprioritising the second aspect of PCC. A characteristic of this practice pattern was that GPs tried to make a patient’s view about medicines align with the GP’s view. GPs using this pattern portrayed discomfort with the uncertainties of medicine, adhering rigidly to clinical practice guidelines. Less experienced GPs described traits of this practice pattern, perceiving they had limited control and may have compensated by trying to convince patients of the right course of action (to take ‘necessary’ medications). For instance, GPs recalled their efforts to deprescribe were often ineffectual, refuted by the patient or another doctor who prescribed the medication. Overall, this could mean that GPs following the ‘Directive’ practice pattern perceived they were unable to fully implement PCC in this context. Instead, GPs emphasised their own understanding of good quality care which was to focus on patient adherence to ‘essential’ medications.

A recent study conducted in the Netherlands focused on decision-making styles of medical specialists, medical residents, nurse practitioners and clinician’s assistants (*n* = 394) [35]. They found that medical residents and participants who were younger (aged less than 35 years old) preferred a clinician-directed approach over a shared approach to decision-making. The authors stated the low proportion of medical residents preferring a SDM approach was “disturbing” [35]. This study did not offer reasons behind this preference, but it was questioned whether medical residents lacked the clinical experience required to perform SDM. This study, and ours, may point to a need for structured mentoring and support for doctors to integrate patient preferences with clinical decisions, specifically for prescription management in older patients.

‘Goal-oriented’, the second practice pattern, aligned with what we would recognise as PCC. GPs who conformed to this pattern utilised their communication skills and clinical acumen to deviate from guidelines and to make decisions based on patient preferences. These GPs meet the fundamental aspects of PCC outlined above and GPs using this practice pattern had a shared approach to decisions. However, some GPs assumed all patients wanted to participate in SDM without establishing the patient’s actual preference or how capable that patient felt to be involved. Our previous qualitative study showed there are older people who preferred to defer decisions about medicines to their doctor or companion [10]. It is understandable that not all patients value being an active participant in decision-making as SDM can be challenging and burdensome. Instead of encouraging patients to emulate an ‘ideal’ or active patient, allowing delegation respects a patient’s wishes, while supporting them to flourish to the best of their ability. This would still be considered SDM and a patient-centred approach to optimising medicines [4, 21].

‘Tacit’, the third practice pattern GPs displayed traits of, patient goals and preferences were used in an intrinsic way. Lengthy relationships with their older patients combined with clinical expertise in complex medical situations led GPs to believe they could recognise what an individual patient valued; this could then inform the GP about the right thing to do. Prima facie, one might expect that GPs using the ‘Tacit’ practice pattern may not see PCC as essential to provide optimal care for older patients. However, this practice pattern, overall, did have some aspects of PCC. Including treating patients with respect and compassion by allocating enough time in consultations, understanding goals and preferences held by older patients in general, and taking care to avoid unnecessary risks that could negatively impact a patient’s life.

It could be posited that the general goals identified by GPs using the ‘Tacit’ practice pattern may be right for some older patients. A study conducted in the US asked older patients with multi-morbidities (*n* = 357) to rank pre-determined health outcomes [36]. 76% identified independence as their most important goal (followed by symptom relief, pain management and staying alive) – all of which were goals mentioned by GPs using the ‘Tacit’ practice pattern. However, individual patients’ goals and preferences are not stable, they change with circumstances, worsening health conditions and may vary based on the decision to be made [37, 38]. Importantly, in most cases individual patients are best placed to inform about their specific goals and preferences [39, 40].

However, translating general health goals into specific decisions to be made and communicating with patients about this is not easy. Being responsive to a patient’s unique identity and subjective experience would involve eliciting their goals and preferences in every day clinical practice, which GPs with the ‘Tacit’ practice pattern tended not to do. Even though it appeared that GPs using this practice pattern had the confidence and experience to deviate from clinical guidelines to change a medication regimen to align with their patient’s priorities this was usually initiated by patients.

Regarding implementing PCC that supports patients to be as autonomous as possible in decision-making, GPs working in the ‘Tacit’ and ‘Directive’ practice patterns tended to think that most older patients prefer not to be involved in decisions about their health. With this assumption, GPs might not have been responsive to a patient’s unique identity and subject experience – the second fundamental element of PCC. In general, clinicians tend to underestimate their patient’s preferred level of involvement in decisions [41] or they do not check the patient’s preferred level of involvement [42]. Tailoring to the differences in these practice patterns could inform the implementation of interventions such as decision support tools, goal elicitation strategies and conversation guides to support shared decision-making about medicines [43]. Of note, it is not simple to harmonise the clinical aspects of medicine with patient priorities or to integrate discordant patient-GP-companion goals. A study focused on embedding patient priorities into clinical decision-making reported how difficult it was for clinicians to move away from disease-specific guidelines towards patients’ priorities – even when patients’ priorities were known to clinicians [5]. Shared decision-making in the context of older adults and polypharmacy is challenging: first eliciting patients’ goals and preferences and then combining this knowledge with medical decisions – while considering the preferred level of involvement of the patient. This requires exceptional skill (in medicine and communication), creativity and courage to embrace uncertainty and make decisions beyond clinical practice guidelines.

## Conclusions

This study identified three practice patterns that GPs follow in the context of patients’ goals and preferences in managing medicines. Although some prior research demonstrated differences in how GPs approach shared decision-making, as a whole they had not shown how these differences were operationalised in the context of older adults taking multiple medicines – as this study has. Importantly, these practice patterns were more or less supportive of PCC and there were some factors that influenced how patterns were distributed, according to GP experience. If PCC is to be recommended for older patients taking multiple medicines and active elicitation of patients’ goals and preferences is central to this process, support for early career GPs is imperative to achieve this. For all GPs, it is important to appreciate the complex, often unrelenting work of caring for older patients with complicated health situations; and to reiterate the need to elicit individual patients’ goals, even if a GP thinks they may have ‘seen it all before’. Future research could look towards practical strategies that support elicitation of patients’ goals and preferences and their integration into clinical decisions to achieve goal-concordant PCC. Understanding how clinicians operationalise PCC, SDM and deprescribing in clinical practice could inform future research on how to facilitate patient involvement and help patients to achieve their goals.

## Supplementary Information

**Additional file 1.** Appendix A. COREQ checklist

## Data Availability

Due to restrictions imposed by the ethics committee, we will not be able to provide a de-identified data set. This is because participants did not consent to have their data made publicly available.

## Abbreviations

GPs: General practitioners
PCC: Person/patient centred care
SDM: Shared decision-making
RACGP: Royal Australian College of General Practitioners
COREQ: Consolidated criteria for reporting qualitative research

## References

[1] Kuijpers MAJ, van Marum RJ, Egberts ACG, Jansen PAF. Relationship between polypharmacy and underprescribing. Br J Clin Pharmacol. 2008. 10.1046/j.0306-5251.2007.02961.x. 10.1111/j.1365-2125.2007.02961.x PMC2291281 17578478

[2] Roughead EE, Anderson B, Gilbert AL. Potentially inappropriate prescribing among Australian veterans and war widows/widowers. Intern Med J. 2007. 10.1111/j.1445-5994.2007.01316.x. 10.1111/j.1445-5994.2007.01316.x 17535384

[3] Thomsen LA, Winterstein AG, Sondergaard B, Haugbolle LS, Melander A. Systematic review of the incidence and characteristics of preventable adverse drug events in ambulatory care. Ann Pharmacother. 2007. 10.1345/aph.1H658. 10.1345/aph.1H658 17666582

[4] Jansen J, Naganathan V, Carter SM, McLachlan AJ, Nickel B, Bonner C, et al. Too much medicine in older people? Deprescribing through shared decision making. BMJ. 2016. 10.1136/bmj.i2893. 10.1136/bmj.i2893 27260319

[5] Tinetti ME, Fried TR, Boyd CM. Designing health care for the most common chronic condition—multimorbidity. JAMA. 2012. 10.1001/jama.2012.5265. 10.1001/jama.2012.5265 PMC4083627 22797447

[6] Scott IA, Hilmer SN, Reeve E, Potter K, Le Couteur D, Rigby D, et al. Reducing inappropriate polypharmacy: the process of deprescribing. JAMA Int Med. 2015. 10.1001/jamainternmed.2015.0324. 10.1001/jamainternmed.2015.0324 25798731

[7] Farrell B, Mangin D (2019). Deprescribing is an essential part of good prescribing. Am Fam Physician.

[8] Hoffmann T, Jansen J, Glasziou P. The importance and challenges of shared decision making in older people with multimorbidity. PLoS Med. 2018. 10.1371/journal.pmed.1002530. 10.1371/journal.pmed.1002530 PMC5849298 29534067

[9] Reeve E, Shakib S, Hendrix I, Roberts MS, Wiese MD. Review of deprescribing processes and development of an evidence based, patient-centred deprescribing process. Br J Clin Pharmacol. 2014. 10.1111/bcp.12386. 10.1111/bcp.12386 PMC4239968 24661192

[10] Weir K, Nickel B, Naganathan V, Bonner C, McCaffery K, Carter SM, et al. Decision-making preferences and deprescribing: perspectives of older adults and companions about their medicines. J Gerontol B Psychol Sci Soc Sci. 2018. 10.1093/geronb/gbx138. 10.1093/geronb/gbx138 29190369

[11] Stiggelbout AM, Van der Weijden T, De Wit MP, Frosch D, Legare F, Montori VM, et al. Shared decision making: really putting patients at the Centre of healthcare. BMJ. 2012. 10.1136/bmj.e256. 10.1136/bmj.e256 22286508

[12] Epstein RM, Peters E. Beyond information: exploring patients' preferences. JAMA. 2009. 10.1001/jama.2009.984. 10.1001/jama.2009.984 19584351

[13] Petersen AW, Shah AS, Simmons SF, Shotwell MS, Jacobsen JML, Myers AP, et al. Shed-MEDS: pilot of a patient-centered deprescribing framework reduces medications in hospitalized older adults being transferred to inpatient postacute care. Ther Adv Drug Saf. 2018. 10.1177/2042098618781524. 10.1177/2042098618781524 PMC6116773 30181860

[14] Tinetti M, Dindo L, Smith CD, Blaum C, Costello D, Ouellet G (2019). Challenges and strategies in patients’ health priorities-aligned decision-making for older adults with multiple chronic conditions. PLoS One.

[15] Vermunt N, Elwyn G, Westert G, Harmsen M, Olde Rikkert M, Meinders M. Goal setting is insufficiently recognised as an essential part of shared decision-making in the complex care of older patients: a framework analysis. BMC Fam Pract. 2019. 10.1186/s12875-019-0966-z. 10.1186/s12875-019-0966-z PMC6555756 31170920

[16] Bomhof-Roordink H, Gärtner FR, Stiggelbout AM, Pieterse AH (2019). Key components of shared decision making models: a systematic review. BMJ Open.

[17] Bayliss EA, Bonds DE, Boyd CM, Davis MM, Finke B, Fox MH (2014). Understanding the context of health for persons with multiple chronic conditions: moving from what is the matter to what matters. Ann Fam Med.

[18] Montori VM, Brito JP, Murad MH (2013). The optimal practice of evidence-based medicine: incorporating patient preferences in practice guidelines. JAMA..

[19] Weir KR, Bonner C, McCaffery K, Naganathan V, Carter SM, Rigby D, et al. Pharmacists and patients sharing decisions about medicines: development and feasibility of a conversation guide. Res Soc Adm Pharm. 2019. 10.1016/j.sapharm.2018.08.009. 10.1016/j.sapharm.2018.08.009 30172642

[20] Greenhalgh T, Howick J, Maskrey N. Evidence based medicine: a movement in crisis? Br Med J. 2014. 10.1136/bmj.g3725. 10.1136/bmj.g3725 PMC4056639 24927763

[21] Entwistle VA, Watt IS (2013). Treating patients as persons: a capabilities approach to support delivery of person-centered care. Am J Bioeth.

[22] Epstein RM, Street RL (2011). The values and value of patient-centered care. Ann Fam Med.

[23] Legare F, Witteman HO. Shared decision making: examining key elements and barriers to adoption into routine clinical practice. Health Aff. 2013. 10.1377/hlthaff.2012.1078. 10.1377/hlthaff.2012.1078 23381520

[24] Gillespie RJ, Harrison L, Mullan J. Deprescribing medications for older adults in the primary care context: a mixed studies review. Health Sci Rep. 2018. 10.1002/hsr2.45. 10.1002/hsr2.45 PMC6266366 30623083

[25] Gravel K, Legare F, Graham ID. Barriers and facilitators to implementing shared decision-making in clinical practice: a systematic review of health professionals' perceptions. Implement Sci. 2006. 10.1186/1748-5908-1-16. 10.1186/1748-5908-1-16 PMC1586024 16899124

[26] Benbassat J, Pilpel D, Tidhar M. Patients' preferences for participation in clinical decision making: a review of published surveys. Behav Med. 1998. 10.1080/08964289809596384. 10.1080/08964289809596384 9695899

[27] Chodosh J, Petitti DB, Elliott M, Hays RD, Crooks VC, Reuben DB, et al. Physician recognition of cognitive impairment: evaluating the need for improvement. J Am Geriatr Soc. 2004. 10.1111/j.1532-5415.2004.52301.x. 10.1111/j.1532-5415.2004.52301.x 15209641

[28] Mosher HJ, Lund BC, Kripalani S, Kaboli PJ (2012). Association of health literacy with medication knowledge, adherence, and adverse drug events among elderly veterans. J Health Commun.

[29] Gillespie R, Mullan J, Harrison L. Attitudes towards deprescribing and the influence of health literacy among older Australians. Prim Health Care Res Dev. 2019. 10.1017/S1463423618000919. 10.1017/S1463423618000919 PMC8060829 32799987

[30] Bernacki RE, Block SD (2014). American College of Physicians High Value Care Task Force. Communication about serious illness care goals: a review and synthesis of best practices. JAMA Intern Med.

[31] Austin CA, Mohottige D, Sudore RL, Smith AK, Hanson LC (2015). Tools to promote shared decision making in serious Illness: A systematic review. JAMA Intern Med.

[32] Muscat DM, Shepherd HL, Hay L, Shivarev A, Patel B, McKinn S (2019). Discussions about evidence and preferences in real-life general practice consultations with older patients. Patient Educ Couns.

[33] Weir KR, Naganathan V, Rigby D, McCaffery K, Bonner C, Trevana L, et al. Home medicines reviews: a qualitative study of GPs’ experiences. Aust J Prim Health. 2019. 10.1071/PY19072. 10.1071/PY19072 31733660

[34] Ritchie J, Lewis J, McNaughton Nicholls C, Ormston R (2013). Qualitative research practice: a guide for social science students and researchers.

[35] Driever EM, Stiggelbout AM, Brand PLP (2020). Shared decision making: physicians’ preferred role, usual role and their perception of its key components. Patient Educ Couns.

[36] Fried TR, Tinetti ME, Iannone L, O’Leary JR, Towle V, Van Ness PH. Health outcome prioritization as a tool for decision making among older persons with multiple chronic conditions. Arch Intern Med. 2011. 10.1001/archinternmed.2011.424. 10.1001/archinternmed.2011.424 PMC4036681 21949032

[37] Fried TR, O'Leary J, Van Ness P, Fraenkel L (2007). Inconsistency over time in the preferences of older persons with advanced illness for life-sustaining treatment. J Am Geriatr Soc.

[38] Mulley AG, Trimble C, Elwyn G (2012). Stop the silent misdiagnosis: patients’ preferences matter. BMJ.

[39] Casper GR, Brennan PF (1993). Improving the quality of patient care: the role of patient preferences in the clinical record. Proceedings of the American Medical Informatics Association Annual Symposium on Computer Application in Medical Care.

[40] Little P, Everitt H, Williamson I, Warner G, Moore M, Gould C (2001). Observational study of effect of patient centredness and positive approach on outcomes of general practice consultations. BMJ.

[41] Sonntag U, Wiesner J, Fahrenkrog S, Renneberg B, Braun V, Heintze C (2012). Motivational interviewing and shared decision making in primary care. Patient Educ Couns.

[42] Menear M, Garvelink MM, Adekpedjou R, Perez MMB, Robitaille H, Turcotte S (2018). Factors associated with shared decision making among primary care physicians: findings from a multicentre cross-sectional study. Health Expect.

[43] Légaré F, Stacey D, Turcotte S, Cossi MJ, Kryworuchko J, Graham ID, et al. Interventions for improving the adoption of shared decision making by healthcare professionals. Cochrane Database Syst Rev. 2014;(9)CD006732:1–148. 10.1002/14651858.CD006732.pub3 25222632

