# Supplementary material for: The role of older patients’ goals in GP decision-making about medicines: a qualitative study
Source: BMC Fam Pract. 2021 Jan 8;22:13. doi: 10.1186/s12875-020-01347-y (PMC7796626; doi:10.1186/s12875-020-01347-y)
Supplement: Supplementary file 1 — Additional file 1. Appendix A. COREQ checklist [file 12875_2020_1347_MOESM1_ESM.docx]

**Appendix A. COREQ checklist**

**The Consolidated Criteria for Reporting Qualitative Studies (COREQ): 32-item checklist**

(Table developed from Tong et al., 2007)

| **No. Item** | **Guide questions/description** | **Notes** |
| --- | --- | --- |
| **Domain 1: Research team and reﬂexivity** | | |
| **The research team**  The multidisciplinary research team included experts in qualitative methodology (KW, JJ, KMcC, CB, SC) psychology (JJ, KMcC, CB), pharmacy (AM, DR), general practice (LT, CT), geriatric medicine (VN), epidemiology (LI), ethics (SC), statistics (KMcG) and consumer representative (JC). | | |
| ***Personal Characteristics*** |  |  |
| 1. Interviewer/ facilitator | Which author/s conducted the interview or focus group? | Interviews were facilitated by the first author (KW) |
| 2. Credentials | What were the researcher’s credentials? E.g. PhD, MD | KW: BSci, MPH |
| 3. Occupation | What was their occupation at the time of the study? | At the time of the interviews, KW was a PhD candidate |
| 4. Gender | Was the researcher male or female? | The interviewer (KW) is female |
| 5. Experience and training | What experience or training did the researcher(s) have? | KW was trained in qualitative methods |
| ***Relationship with participants*** | | |
| 6. Relationship established | Was a relationship established prior to study commencement? | KW did not have any contact with participants prior to organising the time for the interview |
| 7. Participant knowledge of the interviewer | What did the participants know about the researcher? e.g. personal goals, reasons for doing the research | Participants were informed that the researcher was interested in exploring GPs’ experiences of medicines management as part of her PhD |
| 8. Interviewer characteristics | What characteristics were reported about the inter viewer/facilitator? e.g. Bias, assumptions, reasons and interests in the research topic | Participants were informed that, as part of her PhD, the researcher was interested in interviewing GPs about their views on the role of patient goals and preferences in medicines management including deprescribing and GPs’ experiences of medication reviews |
| **Domain 2: Study design** | | |
| ***Theoretical framework*** | | |
| 9. Methodological orientation and Theory | What methodological orientation was stated to underpin the study? e.g. grounded theory, discourse analysis, ethnography, phenomenology, content analysis | Methods in this study were based on phenomenology and framework analysis. Phenomenological methodology focuses on individual experience |
| ***Participant selection*** | | |
| 10. Sampling | How were participants selected? e.g. purposive, convenience, consecutive, snowball | Purposeful sampling to obtain a representative group in terms of age, gender and location. Subsequent active ‘snowballing’ was used to access hard to reach participant demographics |
| 11. Method of approach | How were participants approached? e.g. face-to-face, telephone, mail, email | Participants were approached using advertisements via the newsletters and email lists of GP organisations (RACGP and Primary Health Networks), through publicly available information (email invitation), in social media, and at medical conferences. Rural GPs were accessed by phoning practice managers and through colleagues |
| 12. Sample size | How many participants were in the study? | There were 32 participants in the study |
| 13. Non-participation | How many people refused to participate or dropped out? Reasons? | There were no participants who dropped out after consenting to participate |
| ***Setting*** |  |  |
| 14. Setting of data collection | Where was the data collected? e.g. home, clinic, workplace | Interviews were conducted over the phone/teleconference or face-to-face at the GP’s medical practice as preferred by the participant |
| 15. Presence of non-participants | Was anyone else present besides the participants and researchers? | Only the participant and researcher (KW) were present at the time of the interview |
| 16. Description of sample | What are the important characteristics of the sample? e.g. demographic data, date | Interviews were conducted between February and October 2018. See Table 1 for demographic characteristics |
| ***Data collection*** |  |  |
| 17. Interview guide | Were questions, prompts, guides provided by the authors? Was it pilot tested? | Interviews were semi-structured. The interview guide was pilot tested with 2 GPs and 1 qualitative research expert |
| 18. Repeat interviews | Were repeat inter views carried out? If yes, how many? | No repeat interviews were carried out |
| 19. Audio/visual recording | Did the research use audio or visual recording to collect the data? | All interviews were audio-recorded |
| 20. Field notes | Were ﬁeld notes made during and/or after the interview or focus group? | Field notes were made throughout the interviews and salient themes were identified during preliminary analysis to help inform the analysis framework |
| 21. Duration | What was the duration of the interviews or focus group? | Interviews lasted between 24 and 55 minutes |
| 22. Data saturation | Was data saturation discussed? | Preliminary analysis during data collection suggested thematic consistency indicating saturation of key themes |
| 23. Transcripts returned | Were transcripts returned to participants for comment and/or correction? | Transcripts were not returned to participants for comment and/or correction |
| **Domain 3: Analysis and findings** | | |
| ***Data analysis*** | | |
| 24. Number of data coders | How many data coders coded the data? | One researcher coded the entire dataset (KW), with a subset (10%) double coded by a second researcher (IB) |
| 25. Description of the coding tree | Did authors provide a description of the coding tree? | No |
| 26. Derivation of themes | Were themes identified in advance or derived from the data? | Themes were derived from the data |
| 27. Software | What software, if applicable, was used to manage the data? | Microsoft Excel was used for Framework analysis |
| 28. Participant checking | Did participants provide feedback on the findings? | One participant did, providing a dual perspective as both a participant and researcher |
| ***Reporting*** | | |
| 29. Quotations presented | Were participant quotations presented to illustrate the themes/ findings? Was each quotation identified? E.g. participant number | Participant quotes from various participants are presented to illustrate themes. All quotes are de-identified with gender and years’ of experience as a GP |
| 30. Data and findings consistent | Was there consistency between the data presented and the findings? | The quotes and themes presented are consistent with the findings described |
| 31. Clarity of major themes | Were major themes clearly presented in the findings? | Major themes are presented clearly under subheadings |
| 32. Clarity of minor themes | Is there a description of diverse cases or discussion of minor themes? | Diverse cases and minor subthemes are discussed after each major theme is described |
